# Pluronic F-127-functionalized magnetic ternary hybrid nanoparticles for co-delivery of paclitaxel and psoralen: a synergistic strategy to overcome multidrug resistance in triple-negative breast cancer

**DOI:** 10.3389/fphar.2026.1952492

**Published:** 2026-10-21

**Authors:** Qian Liu, Fengjie Liu, Panpan Wang, Uladzislau E. Aharodnikau, Mulazim Hussain Asim, Manling Du, Qianwen Li, Mengjie Xie, Zhenjiang Yang, Hong Hu, Yu Cai

**Affiliations:** 1 State Key Laboratory of Bioactive Molecules and Druggability Assessment, Jinan University, Guangzhou, Guangdong, China; 2 International Cooperative Laboratory of Traditional Chinese Medicine Modernization and Innovative Drug Development of Ministry of Education (MOE) of China, Guangzhou, Guangdong, China; 3 Guangdong Key Lab of Traditional Chinese Medicine Informatization, Guangzhou, Guangdong, China; 4 International Science and Technology Cooperation Base of Guangdong Province, Guangzhou, Guangdong, China; 5 School of Pharmacy, Jinan University, Guangzhou, Guangdong, China; 6 School of Traditional Chinese Medicine, Jinan University, Guangzhou, Guangdong, China; 7 Department of Chinese Medicine, The First Affiliated Hospital of Jinan University, Guangzhou, China; 8 Research Institute for Physical Chemical Problems of the Belarusian State University, Minsk, Belarus; 9 Educational-Scientijfic-Production Republican Unitary Enterprise “UNITEHPROM BSU”, Minsk, Belarus; 10 College of Pharmacy, University of Sargodha, Sargodha, Pakistan; 11 Department of Oncology and Hematology, Shenzhen Traditional Chinese Medicine Hospital, Shenzhen, China; 12 Division of Breast Surgery, Department of General Surgery, Shenzhen People’s Hospital (The First Affiliated Hospital, Southern University of Science and Technology; The Second Clinical Medical College, Jinan University), Shenzhen, China

**Keywords:** magnetic ternary hybrid nanoparticles, PLGA-lecithin composite, pluronic F-127 (PEO-PPO-PEO), superparamagnetic iron oxide nanoparticles (SPIONs), triple-negative breast cancer

## Abstract

A series of magnetic ternary hybrid nanoparticles were synthesized through the chemical integration of superparamagnetic iron oxide nanoparticles (SPIONs), poly (lactic-co-glycolic acid) (PLGA), and lecithin, further functionalized with Pluronic F-127 (PEO-PPO-PEO). The ternary architecture afforded nanoparticles with well-defined spherical morphology, tunable particle sizes (69–166 nm), and high colloidal stability with zeta potentials exceeding −30 mV. Spectroscopic analyses, including FTIR, DSC, XRD and PLM, confirmed the successful incorporation of therapeutic agents within the ternary matrix, revealing their amorphous dispersion and strong intermolecular interactions with the carrier components. Magnetic characterization demonstrated retained superparamagnetic behavior, highlighting the magnetic responsiveness of the ternary system. *In vitro* studies against paclitaxel-resistant SUM159/TXR triple-negative breast cancer cells showed that the ternary nanoparticles enhanced cellular uptake, induced synergistic G0/G1 and G2/M cell cycle arrest, and promoted early-stage apoptosis. Co-encapsulation of psoralen and paclitaxel within the ternary hybrid framework amplified cytotoxic effects compared to individual formulations. *In vivo* evaluation using a xenograft tumor model confirmed superior tumor growth inhibition and minimal systemic toxicity. These results underscore the chemical design and structural optimization of Pluronic F-127-functionalized magnetic ternary hybrid nanoparticles as a promising strategy to overcome multidrug resistance and enhance therapeutic efficacy in aggressive cancers. This study constructed an Pluronic F-127-functionalized magnetic ternary hybrid delivery system co-loaded with superparamagnetic nanoparticles, PTX and PSO, achieving synergistic effects of magnetic targeting, hemotherapy and drug-resistance reversal, which provides a novel strategy for precise therapy of drug-resistant TNBC.

## 1 Introduction

A highly aggressive breast cancer subtype, TNBC, constitutes 10%–20% of newly diagnosed cases and is pathologically characterized by the negative expression of three key receptors: estrogen receptor (ER), progesterone receptor (PR), and human epidermal growth factor receptor 2 (HER-2) (Kim et al., 2025; Bray et al., 2024). Owing to the paucity of specific molecular targets for intervention, chemotherapy has remained the primary therapeutic modality for this disease to date.

As a first-line core agent in systemic chemotherapy for TNBC, PTX stabilizes tubulin to block microtubule depolymerization, arresting mitosis and inducing tumor cell apoptosis with confirmed clinical efficacy (Kim et al., 2025; Martín et al., 2025). However, primary or acquired chemoresistance drastically impairs the clinical efficacy of PTX, emerging as a pivotal factor contributing to treatment failure (Beretta et al., 2025; Alalawy, 2024). Superparamagnetic iron oxide nanoparticles (SPIONs) can be exploited for tumor magnetic targeting (Rezaei et al., 2024; Pawar and Prabhu, 2025). Psoralen exhibits promising potential in reversing tumor chemoresistance (Liu et al., 2024; Meng et al., 2024). Under an external alternating magnetic field, SPIONs can accumulate at tumor sites, which helps improve local drug concentration and reduce off-target damage to normal tissues.

Our research team has accumulated extensive expertise in nanoparticle formulation-mediated delivery of bioactive components derived from traditional Chinese medicines (TCMs), with a specific focus on improving the water solubility and *in vivo* bioavailability of poorly water-soluble TCM bioactive ingredients. The constructed nanodrug delivery systems include polymer-lipid nanoparticles, albumin nanoparticles, and nanoprodrugs, among others (Li et al., 2025a; Li et al., 2025b; Lan et al., 2026; Xie et al., 2024).

In summary, PSO exhibits drug chemoresistance-reversing activity and enhances the chemosensitivity of TNBC cells to PTX. Meanwhile, SPIONs-mediated magnetic hyperthermia therapy can directly kill drug-resistant tumor cells through local thermal effects and promote the intracellular delivery of chemotherapeutic drugs such as PTX. Both strategies offer highly promising combination therapeutic approaches for reversing PTX resistance. Nevertheless, the therapeutic potency of PTX and PSO remains subpar, stemming from their inherent aqueous insolubility and low *in vivo* bioavailability. Against the backdrop of advancing nanomedicine, the encapsulation of TCM bioactive components into nanoparticulate carriers offers a viable avenue to augment their tumor-targeting capacity and optimize therapeutic efficacy.

To date, most reported nanocarriers for paclitaxel-resistant TNBC therapy are limited to magnetic nanoparticles or polymer-lipid nanoparticles (PLNs) alone for drug delivery. For instance, several studies have utilized bare or polymer-coated Fe_3_O_4_-based magnetic nanoparticles to deliver chemotherapeutic agents (Al-Obaidy et al., 2023), while other investigations have constructed PLN-based nanoplatforms to encapsulate PTX (Salel and Iyisan, 2023). Nevertheless, few studies have rationally encapsulated oleic acid-functionalized superparamagnetic iron oxide nanoparticles together with paclitaxel and PSO within a single PLN matrix to achieve synergistic reversal of TNBC multidrug resistance. Different from conventional single-component or single-drug delivery systems, our ternary hybrid PLNs integrate magnetic responsiveness, dual-drug co-delivery, and combinatorial anti-resistant effects, thereby presenting unique structural and therapeutic novelty for TNBC therapy.

Based on this, we constructed a composite lipid magnetic nanoparticle system, in which SPIONs, PSO, and PTX were encapsulated using polymeric lipids. Meanwhile, this system was compared with P/P-PLNs and P/P-IOPNs.

This study is the first to integrate three functional components—superparamagnetic nanoparticles (with both MRI imaging and magnetic targeting functions), PTX (a first-line chemotherapeutic agent for triple-negative breast cancer), and PSO (a drug-resistance reversal agent)—into a single PLNs carrier, constructing an integrated delivery system with three core functions: magnetic targeting enrichment, chemotherapeutic cytotoxicity, and drug-resistance reversal.

Compared with traditional single-drug-loaded nanosystems (carrying only chemotherapeutic agents or drug-resistance reversal agents), the core innovation of this study lies in breaking the limitation of single-function carriers. Rather than simple physical combination of components, combined functional effects is achieved via carrier optimization: superparamagnetic nanoparticles enable magnetic targeting enrichment at tumor sites and reduce adverse effects on normal tissues; PTX exerts chemotherapeutic effects to kill tumor cells; PSO effectively reverses tumor resistance to chemotherapy drugs. Combined action of the three components addresses the key drawbacks of conventional chemotherapy, including drug resistance, severe systemic toxicity, and poor targeting capability.

## 2 Materials and methods

### 2.1 Materials

FeCl_2_·4H_2_O, Ammonia, sodium oleate, and soybean lecithin were from Macklin Biochemical Co., Ltd. (Shanghai, China). FeCl_3_·6H_2_O was obtained from Aladdin Biochemical Technology Co., Ltd. (Shanghai, China). Pluronic F-127 (PF-127) was supplied by Yuanye Biotechnology Co., Ltd. (Shanghai, China). PSO was acquired from Chengdu Herpurify Co., Ltd. (Chengdu, China). PTX was purchased from Dalian Meilun Biotechnology Co., Ltd. (Dalian, China). Poly (lactic-co-glycolic acid) (PLGA) and 1,2-Distearoyl-sn-glycero-3-phosphoethanolamine-poly (ethylene glycol) 2000 (DSPE-PEG_2000_) were obtained from Xi’an Ruixi Biotechnology Co., Ltd. (Xi’an, China). Tween-80 was supplied by Hubei Gedian Humanwell Pharmaceutical Excipients Co., Ltd. (Ezhou, China). Chloroform was purchased from Guangzhou Chemical Reagent Factory (Guangzhou, China), and anhydrous ethanol was obtained from Tianjin Yongda Chemical Reagent Co., Ltd. (Tianjin, China). Acetonitrile was supplied by Beijing Mairead Technology Co., Ltd. (Beijing, China). Coumarin 6 (C6) was acquired from Tokyo Chemical Industry Co., Ltd. (Tokyo, Japan).

Fetal bovine serum (FBS) was from Sai’ao Mei Cell Technology Co., Ltd. (Beijing, China). Dulbecco’s Modified Eagle Medium (DMEM) was obtained from Merck (Suzhou, China). Penicillin/streptomycin and 0.25% Trypsin-EDTA (1×) were purchased from Life Technologies Corporation (USA). Insulin was acquired from Yeasen Biotechnology Co., Ltd. (Shanghai, China). Cell Counting Kit-8 (CCK-8) solution was supplied by Kefan Biotechnology Co., Ltd. (Guangzhou, China).

### 2.2 Animals and cell lines

Female BALB/c nude mice (5–6 weeks, 18–20 g) were provided by the Guangdong Provincial Center for Laboratory Animals. All animal experiments were approved by the Institutional Animal Care and Use Committee of Jinan University (Approval No.: IACUC-20250924-10).

The cell line SUM159PT and SUM159/TXR was obtained from the Cancer Research Institute of Jinan University. The cells were cultured in F-12 medium supplemented with 9% FBS, 1% penicillin/streptomycin, 0.1% hydrocortisone, 0.05% insulin and 1% HEPES buffer, under the conditions of 37 °C and 5% CO_2_.

### 2.3 Preparation of nanoparticles

#### 2.3.1 Preparation of SPIONs@OA

Precisely weighed 0.966 g FeCl_2_•4H_2_O and 1.551 g FeCl_3_•6H_2_O were dispersed in 50 mL deoxygenated water under stirring to form Solution A (Wulandari et al., 2022). Separately, 7 g concentrated ammonia solution (25%–28%) was diluted with 25 mL purified water to prepare Solution B, and 1.5 g sodium oleate was ultrasonicated in 50 mL purified water to obtain Solution C. Solution B was slowly added to Solution A; the mixture reacted at 40 °C for 30 min and crystallized at 60 °C for 1 h. The crude SPIONs were magnetically separated, rinsed with purified water 2–3 times to neutrality, yielding a suspension. Solution C was then added to the suspension, stirred at 50 °C for 30 min, and the product was magnetically separated and rinsed again. After centrifugation at 13,900 rpm for 1 h, the precipitate was vacuum-dried to obtain SPIONs@OA.

#### 2.3.2 Preparation of P/P-IOPNs

##### 2.3.2.1 Preparation of PF-127-modified SPIONs

A 0.5 g portion of SPIONs@OA nanoparticles was accurately weighed and dispersed in 100 mL of 1% PF-127 aqueous solution. The air inside the system was replaced with nitrogen, and the mixture was stirred at room temperature for 12 h. Post stirring, the magnetic nanoparticles were magnetically isolated, washed 2–3 times with 10 mL of deoxygenated water each time, centrifuged at 13,900 rpm for 1 h, and the precipitate was collected and vacuum-dried to obtain PF-127-modified SPIONs, namely P/P-IOPNs.

##### 2.3.2.2 Preparation of P/P-IOPNs

Aqueous Phase Preparation: 100 mg of PF-127-modified SPIONs were weighed and added to an 50 mL of deoxygenated water, with constant stirring to form a homogeneous mixture.

Organic Phase Preparation: 5 mg of PTX was weighed and dissolved in 1.5 mL of chloroform via ultrasonication; after complete dissolution, 5 mg of PSO was added and further dissolved by ultrasonication.

Solvent Evaporation: The organic phase was gradually introduced into the aqueous phase at 30 °C, with sustained stirring for 6 h to facilitate solvent evaporation.

Sterile Filtration: The suspension was filtered via a 0.22 μm polyethersulfone (PES) filter to obtain P/P-IOPNs.

#### 2.3.3 Preparation of P/P-PLNs

Aqueous Phase Preparation: 80 mg of soybean lecithin and 20 mg of DSPE-PEG_2000_ were weighed out and fully dissolved in 0.3 mL of anhydrous ethanol. Subsequent to dissolution, 50 mL of purified water and 0.2 mL of Tween-80 was added. The resultant mixture was stirred thoroughly and incubated at 2 °C–8 °C.

Organic Phase Preparation: 100 mg of PLGA was accurately weighed and fully dissolved in 1.5 mL chloroform via ultrasonic treatment. Following dissolution, 5 mg PTX was added and further dispersed by ultrasonication. Once PTX was fully dissolved, 5 mg PSO was added and dissolved using the same ultrasonic approach.

Emulsification: Under the action of a probe ultrasonic processor, the organic phase was gradually added into the aqueous phase. The sonication power was set at 30%, with a total sonication time of 5 min. The pulse setting was 4 s on and 1 s off. The temperature of the solution was maintained at 2 °C–15 °C during sonication.

Organic Solvent Removal: Organic solvent was removed by evaporation at 45 °C for a 6 min duration.

Sterile Filtration: The suspension was filtered via a 0.22 μm polyethersulfone (PES) filter to obtain P/P-PLNs.

#### 2.3.4 Preparation of P/P-TCNs

Aqueous Phase Preparation: 80 mg of soybean lecithin and 20 mg of DSPE-PEG_2000_ were weighed out and fully dissolved in 0.3 mL of anhydrous ethanol. Subsequent to dissolution, 50 mL of purified water and 0.2 mL of Tween-80 was added. The resultant mixture was stirred thoroughly and incubated at 2 °C–8 °C.

Organic Phase Preparation: 100 mg of PLGA was accurately weighed and fully dissolved in 1.5 mL chloroform by ultrasonic treatment. Following dissolution, 5 mg PTX was added and further dispersed by ultrasonication. Once PTX was fully dissolved, 5 mg PSO was added and dissolved using the same ultrasonic approach. Finally, 100 mg of SPIONs@OA was incorporated and dispersed uniformly.

Emulsification: The organic phase was slowly added to the aqueous phase under ultrasonic probe, followed by ultrasonication at 30% power for 5 min.

Organic Solvent Removal: Organic solvent was removed by evaporation at 45 °C for 6 min.

Sterile Filtration: The suspension was filtered via a 0.22 μm polyethersulfone (PES) filter to obtain P/P-TCNs.

#### 2.3.5 Optimization of P/P-TCNs

A set of experiments based on the single-factor variable principle was carried out herein, where the investigated parameters included phospholipid level, phospholipid-to-modified phospholipid ratio, PLGA level, PSO and PTX level, Tween-80 level, organic phase level, as well as SPIONs@OA level. The experimental results indicated that phospholipid level, PLGA level and Tween-80 level were the three key factors affecting the nanoparticle size and PSO encapsulation efficiency. Subsequently, a three-factor, three-level Box–Behnken design was adopted for formulation optimization. This study selected phospholipid level, PLGA level and Tween-80 level as the core experimental factors for subsequent research, in compliance with the fundamental principles of the Box-Behnken experimental design (Lalchandani et al., 2024). Phospholipid amount, PLGA amount and Tween-80 volume were selected as independent variables. The three levels of phospholipid were set as 50 mg, 100 mg and 150 mg; the three levels of PLGA were 50 mg, 100 mg and 150 mg; and the three levels of Tween-80 were 0.1 mL, 0.2 mL and 0.3 mL. Particle size (Y_1_) and encapsulation efficiency (Y_2_) were chosen as response variables, with minimization of particle size and maximization of encapsulation efficiency as the optimization criteria. This design contained 15 experimental runs, consisting of 12 factorial points and 3 replicated central points to assess experimental error. A second-order polynomial model was applied to fit the obtained experimental data. Analysis of variance (ANOVA) was used to evaluate the significance of model terms and the lack-of-fit. Multi-objective optimization was performed using the desirability function. All statistical calculations were conducted using Design-Expert 13.0 software.

For fluorescence imaging of the three prepared nanoparticles, C6 was loaded into the nanoparticles following the aforementioned preparation procedures to prepare fluorescence-labeled C6-PLNs, C6-IOPNs and C6-TCNs.

### 2.4 Characterization of nanoparticles

The particle size, PDI and zeta potential of PLNs, IOPNs, TCNs, P/P-PLNs, P/P-IOPNs and P/P-TCNs were determined using a Dynamic light scattering particle size analyzer (ZS90, Malvern, UK) with three technical replicates. The samples were stained with phosphotungstic acid and observed under a transmission electron microscope (TECNAI 10, Thermo Fisher Scientific, USA) to evaluate the morphology of the nanoparticles. For TEM observation, one batch of nanoparticles was used. Multiple random fields were captured to characterize particle morphology.

For the determination of PTX and PSO contents in the nanoparticles, HPLC analysis was carried out at 25 °C on a Phenomenex C18 column (250 × 4.6 mm, 4 μm; Agilent, USA) under the following conditions: 10 μL injection volume, acetonitrile-purified water (55:45, v/v) mobile phase, 1.0 mL/min flow rate, and 227 nm detection wavelength. Two parallel samples were tested for each formulation.

Full method validation was executed. The HPLC quantitative method for psoralen (PSO) and paclitaxel (PTX) was systematically validated in terms of specificity, linearity, precision, sample recovery, and solution stability.

For specificity, no chromatographic interference was observed from blank PLNs, IOPNs, TCNs, drug-loaded P/P-PLNs, P/P-IOPNs, P/P-TCNs, free PSO, free PTX, or the PSO/PTX mixture. The resolution (R) between PSO and PTX was greater than 1.5, indicating satisfactory specificity of the established method.

For linearity, the standard curve of PSO was fitted as y = 57.039x – 0.2813 (R^2^ = 1), and the standard curve of PTX was y = 19.7318x – 1.1640 (R^2^ = 1). Both analytes exhibited excellent linear relationships within the concentration range of 1–16 μg/mL.

For precision, low, medium, and high concentrations (1, 4, and 16 μg/mL) were determined with five replicates within 1 day for intra-day precision, and continuously detected for three consecutive days for inter-day precision. The relative standard deviations (RSDs) of intra-day and inter-day precision for PSO and PTX were all less than 2%, demonstrating good precision of the analytical method.

For sample recovery, blank PLNs, IOPNs, and TCNs (1 mL each) were spiked with low, medium, and high concentrations of PSO and PTX (1, 4, and 16 μg/mL). The recoveries of PSO and PTX ranged from 95% to 105%, with all RSD values below 3%, indicating acceptable accuracy and recovery performance.

For solution stability, the RSD values of PSO and PTX within 48 h were 0.79% and 1.55%, respectively, confirming that both analytes remained stable for 48 h under experimental conditions.

Encapsulation efficiency was measured by ultrafiltration using a 30 kDa MWCO membrane, and the EE and DL of PSO and PTX were derived from the respective calculation formulas (Zhang et al., 2023). Two parallel samples were tested for each formulation.

$$\text{EE }\left(\%\right)=\left(\text{Ma }-\text{ Mb}\right)\;/\text{ Ma}\times 100$$

$$\text{DL }\left(\%\right)=\left(\text{Ma}-\text{Mb}\right)\times \text{dilution ratio }/\text{ Me}\times 100$$

where, Ma: Total mass of PSO or PTX, Mb: Mass of free PSO or PTX, Me: Total mass of PSO or PTX + Total mass of carriers.

FTIR spectroscopy analysis was conducted on a Nicolet iS50 spectrometer (Thermo Fisher Scientific, USA), with test samples covering PSO, PTX, their physical mixture, nanoparticle formulations (IOPNs, PLNs, TCNs, P/P-PLNs, P/P-IOPNs, P/P-TCNs), and nanoparticle-drug physical mixtures [(IOPNs + PSO + PTX), (PLNs + PSO + PTX), (TCNs + PSO + PTX)]. The groups of PSO, PTX, their physical mixture, P/P-PLNs, P/P-IOPNs and P/P-TCNs contain equal amounts of PTX or PSO. Similarly, PLNs and P/P-PLNs, IOPNs and P/P-IOPNs, TCNs and P/P-TCNs were prepared with identical level of excipients. FTIR spectroscopy analysis was performed once using one batch of nanoparticle sample.

Individual samples of the PSO-PTX physical mixture, P/P-IOPNs, P/P-PLNs and P/P-TCNs which contain equal amounts of PTX or PSO were prepared for differential scanning calorimetry characterization, which was implemented on a DSC analyzer (TGA/DSC 3+, Mettler Toledo, Switzerland). The experimental parameters were set as follows: scanning temperature interval of 25 °C–600 °C at 10 °C/min heating rate. DSC analysis was performed once using one batch of nanoparticle sample.

PSO + PTX, PLNs, IOPNs, TCNs, P/P-PLNs, P/P-IOPNs and P/P-TCNs were prepared for X-ray diffraction (Rigaku Corporation, Miniflex600) and determined under the conditions of a scanning rate of 5°/min and a scanning range of 5°–90°. They have the same amount of active ingredients. PSO + PTX, P/P-PLNs, P/P-IOPNs and P/P-TCNs were also prepared for polarized light inspection (PLM) (Leica, DM750). X-ray analysis was performed once using one batch of nanoparticle sample.

The same amout powders of SPIONs@OA, IOPNs, TCNs, P/P-IOPNs and P/P-TCNs which contain same level of excipients were placed into plastic vials and sealed. The magnetic properties of the samples were analyzed via a PPMS Dynacool (Quantum Design, USA) at 25 °C within a 20 kOe magnetic field, and saturation magnetization values were obtained from the magnetic hysteresis loops. Magnetic properties was performed once using one batch of nanoparticle sample.

*In vitro* release kinetics of PTX and PSO was assessed by dialysis. P/P-IOPNs (1.50 mL), P/P-PLNs (1.0 mL) and P/P-TCNs (1.0 mL) containing equivalent amounts of PTX and PSO were placed into 10 kDa Molecular Weight Cut-Off dialysis bags, which were then immersed in 50 mL 0.3% Tween-80-containing Phosphate-Buffered Saline (PBS, pH 7.4) and incubated at 37 °C (stirring speed 100 rpm). Three parallel samples were used for the *in vitro* drug release assay. At 1, 2, 4, 8, 12, 24 and 36 h, 1 mL of medium was sampled and new equal volume of fresh PBS was added. After filtration through a 0.22 μm filter, the cumulative release of PTX and PSO was determined via the aforementioned HPLC assay.

### 2.5 Cytotoxicity

*In vitro* cytotoxicity was assessed via the CCK-8 method. SUM159PT paclitaxel-sensitive cells and SUM159/TXR paclitaxel-resistant cells were seeded at 5.0 × 10^3^ cells/well in 96-well plates and cultured for 24 h to achieve adhesion. The cells were then treated with varying concentrations of PSO, PTX, their physical mixture, P/P-IOPNs, SPIONs@PF-127, P/P-PLNs, PLNs, P/P-TCNs and TCNs, with untreated cells serving as controls and blank medium serving as background correction (6 technical replicate wells for each group). Following 48 h of incubation, CCK-8 solution was added and incubation for another 4 h. Absorbance at 450 nm was detected using a ReadMax 1200 full-wavelength microplate reader, and cytotoxicity was computed according to the specified formula.:

Calculation Formulas for Cell Viability and Resistance Index

$$\text{Cell Viability }\left(\%\right)=\left(\text{ODa }-\text{ ODb}\right)\;/\;\left(\text{ODc }-\text{ ODb}\right)\times 100$$

$$\text{Resistance Index }\left(\text{RI}\right)={\text{IC}}_{50}\text{ of SUM}159/\text{TXR }/\;{\text{IC}}_{50}\text{ of SUM}159\text{PT}$$

Where, ODa represents the absorbance of the test sample, ODb represents the absorbance value of the blank well contain only culture medium, ODc represents the absorbance value of the control group which is not treated wtih samples.

### 2.6 *In vitro* cellular uptake

Qualitative Experiment: For comparison of the *in vitro* cellular uptake capacities of P/P-IOPNs, P/P-PLNs and P/P-TCNs in SUM159/TXR cells, 5 × 10^5^ cells per dish were seeded in sterile confocal dishes and incubated for 24 h to allow adhesion. After discarding the original medium, same amout of free C6 and C6-labeled nanoparticles were added to corresponding dishes, with incubation carried out for 1 h, 2 h and 4 h respectively. Post-incubation, cells were rinsed with PBS, fixed with 4% paraformaldehyde, and rinsed again. DAPI staining solution was applied for 10 min of nuclear staining, followed by further rinsing. The cellular uptake test was conducted on a laser scanning confocal microscope (LSM800, ZEISS, Germany).

Quantitative Experiment: SUM159/TXR cells were plated in 6-well plates at 5 × 10^5^ cells/well, and the cell treatment and incubation carried out as per the qualitative assay procedure (3 replicate wells for each group). After incubation, the cell were rinsed with PBS and centrifuged, then resuspended with 500 μL of PBS. Samples were detected on a FACSCanto flow cytometer (BD, USA). The data were analyzed using FlowJo_v10 software.

### 2.7 Cell cycle analysis

SUM159/TXR cells were plated in 6-well plates at 5 × 10^5^ cells/well, treated and incubated as described above (3 replicate wells for each group). After incubation, cells were treated with corresponding drugs and cultured for another 24 h, then harvested by trypsinization, resuspended in pre-cooled 70% anhydrous ethanol, and stored at 4 °C overnight. Following PBS rinsing, cells were resuspended in RNase A reagent, stained with propidium iodide (PI) in the dark at 2 °C–8 °C for 30 min, and analyzed for cell cycle distribution via flow cytometry. The data were analyzed using FlowJo_v10 software.

### 2.8 Cell apoptosis

SUM159/TXR cells were plated in 6-well plates at 5 × 10^5^ cells/well, treated and incubated as detailed above (3 replicate wells for each group). After incubation, cells were treated with corresponding drugs and cultured for another 24 h, then harvested by trypsinization and resuspended in Annexin V Binding Buffer. Annexin V-Fluorescein Isothiocyanate (Annexin V-FITC) and PI reagents were added subsequently, and the mixture was incubated for 30 min. The apoptotic rate was detected by flow cytometry. The data were analyzed using FlowJo_v10 software.

### 2.9 ABCB1 mRNA levels

SUM159/TXR cells were seeded in 6-well plates at 5 × 10^5^ cells/well, treated and incubated as described above (3 replicate wells for each group). After incubation, cells were treated with corresponding drugs and cultured for another 24 h, total cellular RNA was extracted using an RNA extraction kit. The purity and concentration of RNA were determined by spectrophotometry. Subsequently, reverse transcription was performed to synthesize complementary DNA (cDNA). Quantitative real-time PCR was carried out to detect the mRNA expression of ABCB1. GAPDH was used as the internal reference gene. The relative gene expression was calculated by the 2^−ΔΔCt^ method.

### 2.10 *In vivo* biodistribution

To construct a paclitaxel-resistant TNBC xenograft model, SUM159/TXR cells (1 × 10^7^ cells/mouse) were subcutaneously implanted into the axilla of BALB/c nude mice. When tumor volume reached ∼100 mm^3^, mice received 200.0 μL of different formulations via tail vein injection, including NS, free ICG, ICG-PLNs, ICG-IOPNs and ICG-TCNs. Both ICG-IOPNs and ICG-TCNs groups were divided into subgroups with or without external magnetic field exposure. At 1, 2, 4, 8, and 24 h post-injection, animals were anesthetized, and *in vivo* biodistribution was detected by the IVIS Lumina Series III small animal imaging system (PerkinElmer, USA) at excitation of 745 nm and emission of 820 nm.

### 2.11 *In vivo* efficacy

A TNBC resistance model was established with SUM159/TXR cells, all nude mice were randomly divided into 13 groups (6 nude mice per group) and administered the test samples via tail vein injection. The grouping was as follows: NS group, PSO group, PTX group, P/P group, PLNs group, IOPNs group, TCNs group, P/P-PLNs group, P/P-IOPNs group, low-dose P/P-TCNs group, medium-dose P/P-TCNs group, high-dose P/P-TCNs group, and verapamil (Ver) group. The dosage of verapamil was 0.5 mg/kg. For PTX, PSO, PSO + PTX, P/P-PLNs and P/P-IOPNS, the loaded PTX and PSO in nanoparticle formulations were each maintained at a dose of 6 mg/kg (both PSO and PTX) to match the doses of free-drug groups. The low-dose, medium-dose, and high-dose P/P-TCNs groups received PTX and PSO at doses of 3, 6, and 9 mg/kg, respectivley. The dosage of PLNs, IOPNs and TCNs administrated corresponded to the quantity of the blank carrier in P/P-PLNs, P/P-IOPNs and P/P-TCNs. The administration volume of all drugs was 200 μL. A total of 7 doses were given at 3-day intervals. The single and total administered doses of PTX and PSO remained consistent across all groups. Tumor size and mouse body weight were measured and recorded with a vernier caliper before each injection, and tumor volume was computed using Formula 1. On the 22nd treatment day, the nude mice were sacrificed, major organs (heart, liver, spleen, lung anf kidney) were collectd, fixed with 4% paraformaldehyde, stained with hematoxylin and eosin (H&E). Tumor tissues were excised, weighed and imaged, and calculated tumor inhibition rate as Formula 2.

$$\text{Tumor Volume}={\text{ab}}^{2}/2$$ (1)

$$\text{Tumor Inhibition Rate }\left(\%\right)=\left(V_{\text{NS}}-V_{\text{treatment}}\right)\;/\;V_{\text{NS}}\times 100$$ (2)

Where, a represents the Long diameter of the tumor, b represents the Short diameter of the tumor, V_NS_ represents the Tumor volume of nude mice injected with normal saline group, V_treatment_ represents the Tumor volume of nude mice in other groups except the normal saline group.

### 2.12 Statistical analysis

Data are expressed as mean ± SD and statistical analyzed with GraphPad Prism software. The significance of intergroup variations was determined by one-way or two-way ANOVA, and differences were considered statistically significant at P < 0.05.

## 3 Results and discussion

### 3.1 Response surface methodology analysis

Linear regression analysis was performed between the independent variables (phospholipid level, PLGA level, Tween-80 level) and dependent variables (average particle size Y_1_, PSO encapsulation efficiency Y_2_) to evaluate the fitting effect of the model, and the quadratic polynomial regression equation was established as follows:

Y_1_ = 116.4–2.09A + 10.29B + 7.47C − 8.85AB + 0.9750AB − 2.80AC + 3.20BC + 8.79A^2^ + 12.74B^2^ + 7.01C^2^, R^2^ = 0.9948. Within the scope of the experimental design, all variables—covering linear effects (A, B, C), interactive effects (AB, AC) and quadratic effects (A^2^, B^2^, C^2^)—exhibited statistically significant impacts on the response value (p < 0.05).

Y_2_ = 86.08 + 2.15A − 5.62B + 0.6875C − 1.48AB − 0.7300AC + 0.0950BC − 3.10A^2^ − 6.54B^2^ − 4.25C^2^, R^2^ = 0.9981. Within the scope of the experimental design, all variables—covering linear effects (A, B, C), interactive effects (AB, AC) and quadratic effects (A^2^, B^2^, C^2^)—exhibited statistically significant impacts on the response value (p < 0.05).

For the Box-Behnken design, the full ANOVA table containing p-values of linear, interaction and quadratic terms was listed in below Table 1. The model was significant, and the lack-of-fit was non-significant, verifying the reliability of the Box-Behnken model.

**TABLE 1:** Full ANOVA results.

Classification	Items	p-value for average particle size Y1	p-value for PSO encapsulation efficiency Y2
Linear	A-phospholipid level	0.0131	<0.0001
	B-PLGA level	<0.0001	<0.0001
	C-tween 80 level	<0.0001	0.0078
Interaction	AB	0.2687	0.0013
	AC	0.0160	0.0234
	BC	0.0095	0.6922
Quadratic	A^2^	0.0001	<0.0001
	B^2^	<0.0001	<0.0001
	C^2^	0.0004	<0.0001

Design-Expert 13 software was utilized for in-depth analysis of the fitting data, and the corresponding 3D response surface plots for particle size and encapsulation efficiency were constructed as displayed. Figures 1a,g mapped the effects of phospholipid and PLGA levels; Figures 1c,i characterized the combined effects of phospholipid and Tween-80 levels; Figures 1e,k reflected the interactions between PLGA and Tween-80 levels. The pronounced curvature of the surfaces in Figures 1a,e,g,k provided direct visual evidence that PLGA and Tween-80 levels were key factors regulating the mean particle size, and PLGA level was also a critical determinant of encapsulation efficiency.

**FIGURE 1:**
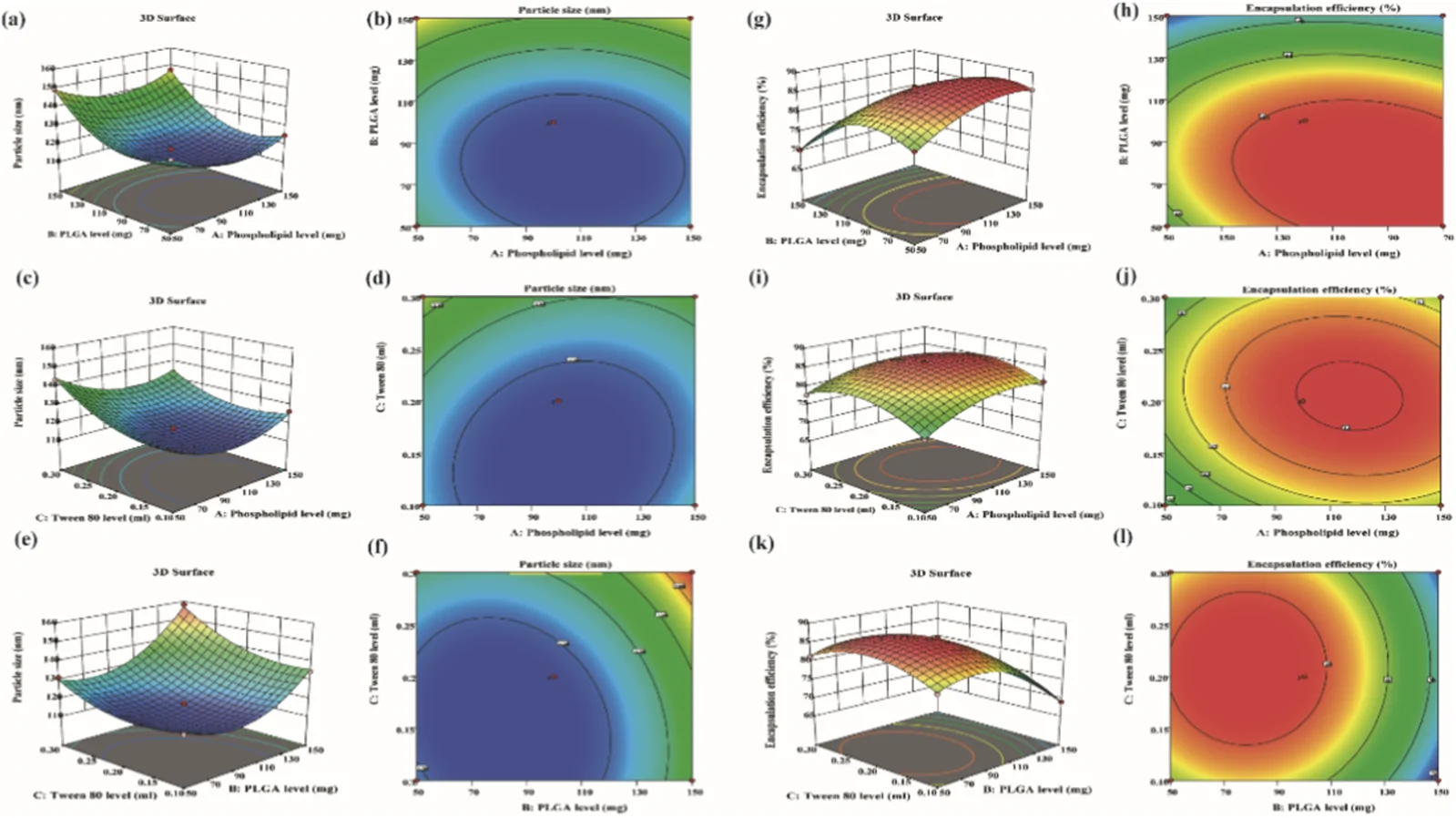
Response Surface Plots of Particle Size and PSO Encapsulation Efficiency of P/P-TCNs. **(a)** 3D response surface plot; **(b)** corresponding contour plot showing the effects of phospholipid level and PLGA level on particle size. **(c)** 3D response surface plot; **(d)** corresponding contour plot showing the effects of phospholipid level and Tween 80 level on particle size. **(e)** 3D response surface plot; **(f)** corresponding contour plot showing the effects of PLGA level and Tween 80 level on particle size. **(g)** 3D response surface plot; **(h)** corresponding contour plot showing the effects of phospholipid level and PLGA level on encapsulation efficiency. **(i)** 3D response surface plot; **(j)** corresponding contour plot showing the effects of phospholipid level and Tween 80 level on encapsulation efficiency. **(k)** 3D response surface plot; **(l)** corresponding contour plot showing the effects of PLGA level and Tween 80 level on encapsulation efficiency.

### 3.2 Nanoparticle characterization

The transmission electron microscopy (TEM) images (Figure 2A) confirmed that all the nanoparticles presented a spherical morphology with uniform dispersion and no aggregation. As shown in Figures 2B,C, the prepared PLNs, IOPNs, TCNs, P/P-PLNs, P/P-IOPNs and P/P-TCNs exhibited desirable particle sizes of 159.2 ± 0.86 nm, 78.4 ± 1.19 nm, 122.3 ± 1.92 nm, 166.5 ± 2.23 nm, 69.07 ± 0.71 nm and 117.2 ± 0.62 nm, respectively; the corresponding PDI were 0.239 ± 0.010, 0.168 ± 0.015, 0.194 ± 0.017, 0.235 ± 0.009, 0.153 ± 0.015 and 0.225 ± 0.012; the zeta potentials were −30.2 ± 0.43 mV, −34.5 ±0.68 mV, −35.1 ± 0.46 mV, −30.4 ± 0.96 mV, −33.4 ±0.60 mV and −37.7 ± 1.57 mV, respectively. The particle sizes of P/P-PLNs, P/P-IOPNs, and P/P-TCNs (69.07–166.5 nm) identified in this study are well-aligned with optimal range, ensuring efficient extravasation through the fenestrations of tumor blood vessels. Additionally, the negative zeta potentials (absolute values > 30 mV) of all nanoparticles indicate strong colloidal stability, which is conducive to maintaining their structural integrity during circulation and further promoting enhanced permeability (Cullion et al., 2024).

**FIGURE 2:**
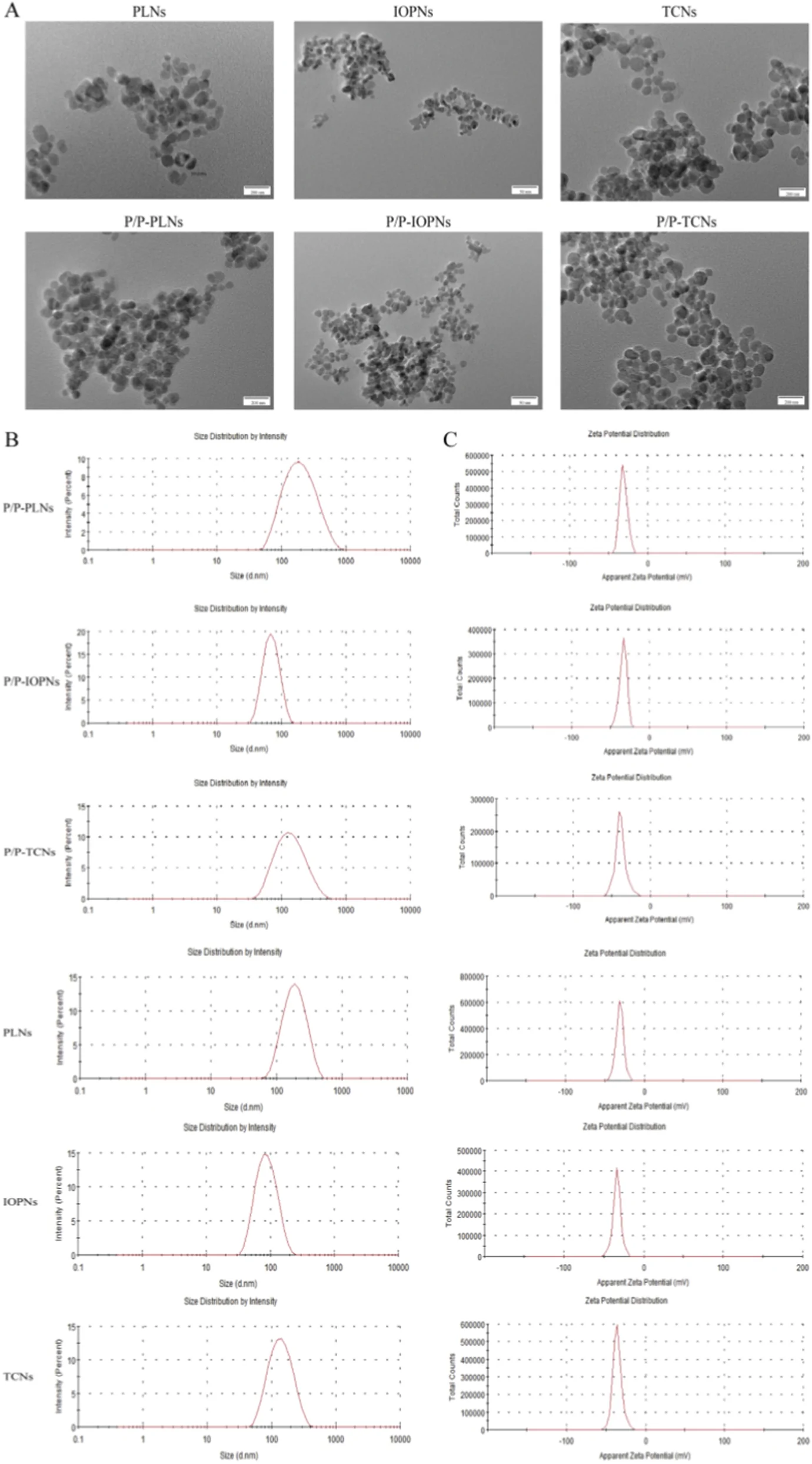
Nanoparticle morphology, particle size and zeta potential. **(A)** Tem images; **(B)** particle size and size distribution; **(C)** zeta potential.

For the prepared P/P-PLNs, P/P-IOPNs and P/P-TCNs, the encapsulation efficiency of PSO was 85.2% ± 1.21%, 77.4% ± 1.32% and 86.3% ± 1.36% respectively; the encapsulation efficiency of PTX was 97.9% ± 1.47%, 93.6% ± 2.13% and 98.7% ± 0.94% respectively; the drug loading of PSO was 1.87% ± 0.12%, 1.32% ±0.09% and 1.93% ± 0.03% respectively; the drug loading of PTX was 1.89% ± 0.06%, 1.44% ± 0.05% and 1.97% ± 0.04% respectively. One-way ANOVA followed by Tukey’s post-hoc test demonstrated that the encapsulation efficiency and drug loading of both PSO and PTX in P/P-IOPNs were significantly lower than those of P/P-PLNs and P/P-TCNs (p < 0.05). No statistically significant differences in these parameters were observed between P/P-PLNs and P/P-TCNs (p > 0.05).

As illustrated in Figure 3ai, a well-defined absorption peak at 1720 cm^-1^ was assigned to the carbonyl (C=O) stretching vibration, a signature characteristic of PSO. In contrast, Figure 3aii exhibited two distinct absorption peaks: the peak at 1720 cm^-1^ was associated with the ester C=O stretching vibration in PTX, while the peak at 1650 cm^-1^ arose from the amide C=O stretching vibration of the drug. Figure 3aiii corresponded to the physical mixture of PSO and PTX, where both characteristic absorption peaks (1720 cm^-1^ and 1650 cm^-1^) were detected, confirming the coexistence of the two drugs in the mixture. Consistently, these two characteristic peaks were also observed in the physical mixtures of IOPNs, PLNs, TCNs with PSO and PTX, see Figures 3av,vii,xi.

**FIGURE 3:**
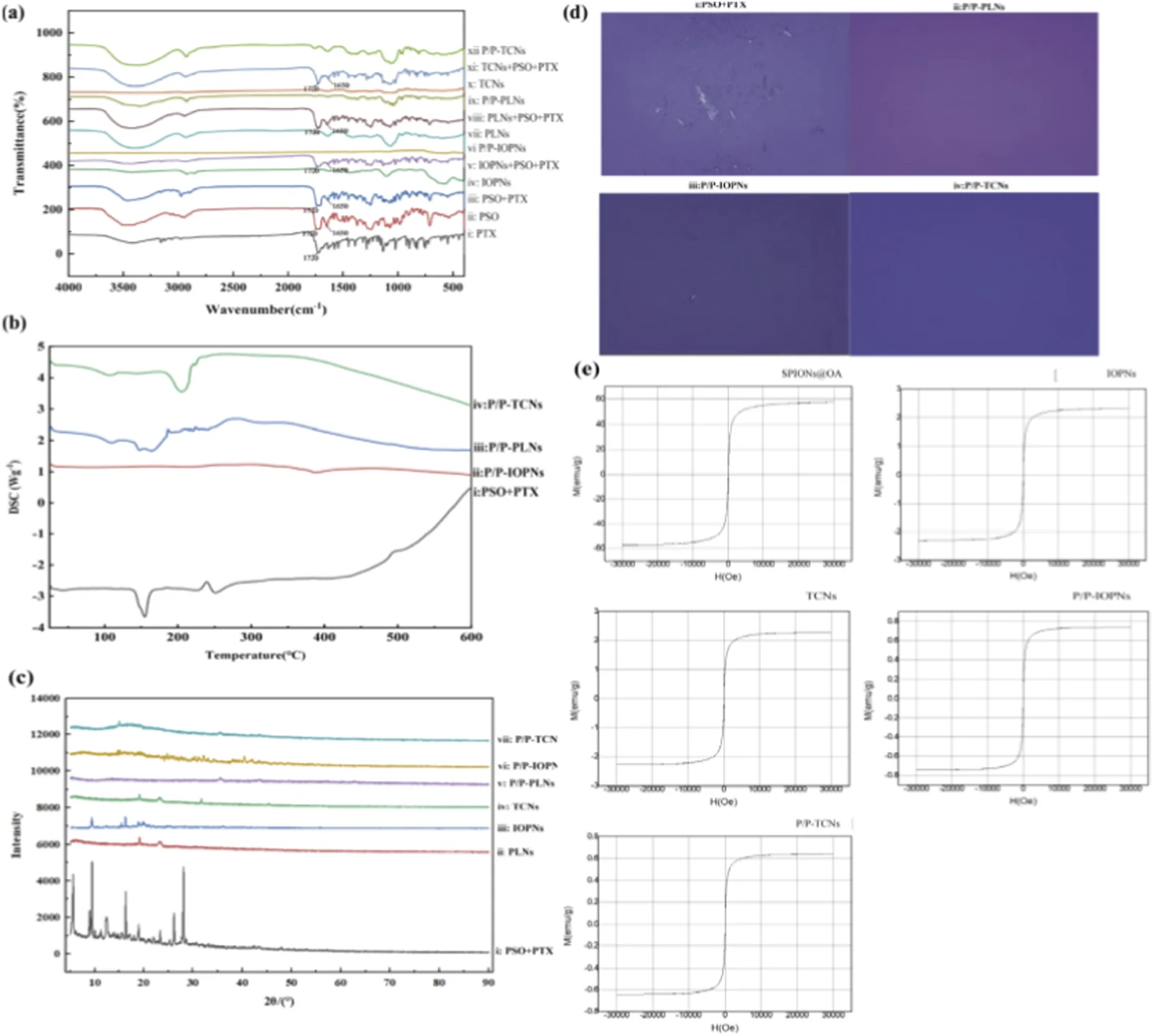
Characterization of nanoparticles. **(a)** Ftir spectra; **(b)** Thermogravimetric analysis; **(c)** XRD Patterns; **(d)** polarized light Mmicroscopy Image; **(e)** magnetic hysteresis loops.

However, these characteristic drug peaks were markedly attenuated or absent in the spectra of drug-loaded nanoparticles, namely P/P-IOPNs, P/P-PLNs and P/P-TCNs (Figures 3avi,ix,xii). The blank carrier spectra (IOPNs, PLNs, TCNs, Figures 3aiv,vii,x) naturally showed no such drug-related peaks, as they contained no PSO or PTX. The attenuation or disappearance of drug characteristic peaks in loaded nanoparticles can be theoretically attributed to the encapsulation of PSO and PTX into the nanoparticles’ hydrophobic core, which restricts the vibrational freedom of the drugs’ C=O groups and weakens their infrared absorption signals; such peak attenuation driven by molecular interactions between drug and carriers (e.g., hydrophobic interactions, hydrogen bonding) is a well-established qualitative indicator of intermolecular association within nanocarrier systems. Notably, FTIR alone cannot verify successful drug encapsulation. Combined with drug loading and encapsulation efficiency, these FTIR results support the incorporation of PSO and PTX into P/P-IOPNs, P/P-PLNs and P/P-TCNs.

To further verify the encapsulated state of PSO and PTX, DSC analysis was conducted on the physical mixture of PSO and PTX, as well as P/P-PLNs, P/P-IOPNs and P/P-TCNs. In Figure 3bi, the DSC thermogram of the PSO + PTX physical mixture exhibited two distinct endothermic peaks at approximately 155 °C and 250 °C, which were attributed to the melting of crystalline PSO and PTX, respectively. In Figure 3bii for P/P-IOPNs, no endothermic peaks corresponding to crystalline PSO or PTX were detected around 155 °C and 250 °C; instead, a new characteristic peak was observed at approximately 388 °C, which might be related to the thermal transition of the inorganic-organic hybrid carrier structure of IOPNs. In sharp contrast as revealed in Figure 3biii, the DSC curve of P/P-PLNs exhibited shifted endothermic peaks at 147 °C and 165 °C, with the complete disappearance of the characteristic crystalline endothermic peaks of PSO (≈155 °C) and PTX (≈250 °C). As for P/P-TCNs in Figure 3bvi, characteristic endothermic peaks were observed at around 108 °C and 205 °C (possibly derived from the thermal behavior of the TCNs carrier), whereas the drug-specific endothermic peaks at 155 °C and 250 °C were absent. Theoretical analysis indicated that the absence of crystalline endothermic peaks of PSO and PTX in the three nanoparticle formulations demonstrated the drugs were no longer in a free crystalline state. On the contrary, the drugs were homogeneously dispersed within the carriers in an amorphous state, or embedded into the internal architecture of the carriers via intermolecular interactions (e.g., hydrogen bonding, hydrophobic interactions)—a phenomenon that impeded the development of ordered crystalline drug domains. By contrast, the PSO-PTX physical mixture exhibited distinct drug-specific endothermic peaks, confirming the drugs existed as independent crystalline phases without interacting with a carrier matrix.

Collectively, these DSC results showed clear differences in endothermic peak profiles between the physical mixture and the three nanoparticle formulations (P/P-PLNs, P/P-IOPNs, P/P-TCNs), verifying the successful encapsulation of PSO and PTX into the nanoparticles, which aligns with the nanocarrier encapsulation mechanisms reported in previous studies.

For a more in-depth investigation into the existing form of PSO and PTX in the nanoparticles, XRD analysis was carried out to identify and characterize the crystalline phases of the test samples.

As shown in Figure 3c, multiple distinct crystalline diffraction peaks were observed in the physical mixture of PSO and PTX, which represents a typical characteristic of crystalline materials. Among these peaks, the strong crystalline diffraction peaks were concentrated in the 2θ range of 5°–30°, corresponding to the intrinsic crystalline planes of PSO and PTX. In contrast, no obvious crystalline diffraction peaks were observed for P/P-PLNs, P/P-IOPNs, and P/P-TCNs, indicating the absence of ordered crystalline domains of PSO and PTX in the nanoparticles. From a theoretical perspective, the absence of characteristic crystalline diffraction peaks of PSO and PTX in P/P-TCNs indicates that the drugs are no longer in a free crystalline state but uniformly dispersed in the carrier matrix in an amorphous or disordered form, which disrupts molecular regularity and eliminates the basis for sharp XRD diffraction peaks (Sarker et al., 2022). Together with FTIR results reflecting drug-carrier intermolecular interactions, these findings further confirm the successful encapsulation of PSO and PTX in the nanoparticles in an amorphous or disordered state, consistent with DSC characterization outcomes.

Polarizing microscope images in Figure 3d also revealed no crystals detected in P/P-PLNs, P/P-IOPNs and P/P-TCNs. These findings are consistent with the XRD results.

The magnetic hysteresis loop is defined as the closed curve that describes the relationship between the applied magnetic field strength H (unit: Oe, ordinate) and the mass magnetization intensity M (unit: emu/g, abscissa) (Dhar and Sabhapandit, 2025).

As shown in Figure 3e, the magnetic hysteresis loops of the prepared SPION@OA, IOPNs, TCNs, P/P-IOPNs and P/P-TCNs all passed through the origin and were centrosymmetric about the origin. These characteristic features demonstrated that the coercivity (H) of all these samples was nearly zero, confirming their excellent superparamagnetic properties. The saturation magnetization intensities of SPION@OA, IOPNs, TCNs, P/P-IOPNs and P/P-TCNs were 57.44, 2.31, 0.64, 2.27 and 0.74 emu/g, respectively. The significant decrease in M values of IOPNs, TCNs, P/P-IOPNs, and P/P-TCNs compared with SPION@OA can be explained by the coating of non-magnetic carrier materials on the surface of SPIONs. The non-magnetic coating layers (e.g., organic polymers, carbon materials) increase the non-magnetic mass fraction of the composite nanoparticles, thereby diluting the magnetic contribution of SPIONs and reducing the overall saturation magnetization. Despite the decrease in M, the retained superparamagnetism of P/P-IOPNs and P/P-TCNs is still sufficient to endow the nanoparticles with magnetic responsiveness (Fahim et al., 2025).

*In vitro* release kineticss of PSO and PTX from P/P-PLNs, P/P-IOPNs and P/P-TCNs were determined in a PBS medium at pH 7.4. As shown in Figures 4a,b, the cumulative release rates of PSO from P/P-PLNs, P/P-IOPNs and P/P-TCNs reached 81.71%, 95.12% and 74.76% within 36 h at pH 7.4, respectively; the corresponding cumulative release rates of PTX were 69.37%, 84.47% and 55.53%, respectively. These results indicated that P/P-TCNs exhibited a certain sustained-release effect compared with P/P-PLNs and P/P-IOPNs at pH 7.4.

**FIGURE 4:**
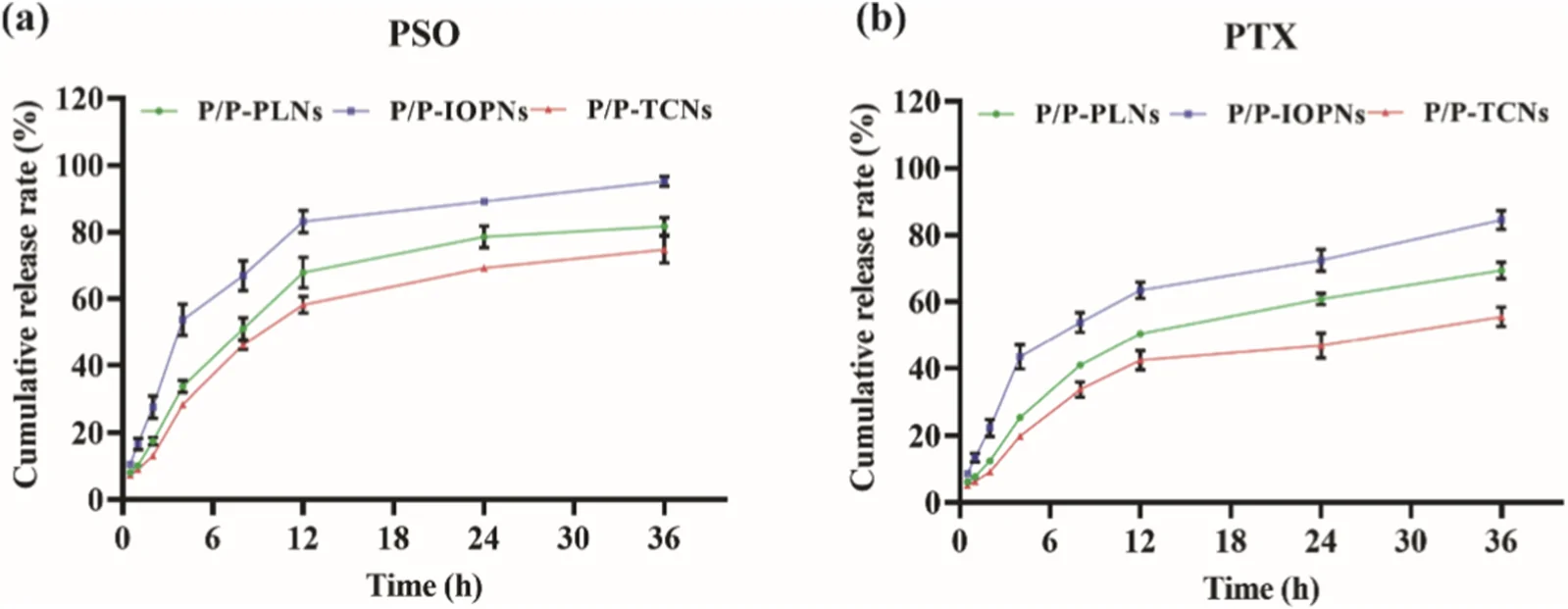
*In Vitro* Drug Release Kinetiscs. **(a)** Release kinetiscs of PSO; **(b)** Release kinetiscs of PTX. Data are expressed as mean ± SD (n = 3).

### 3.3 Cytotoxicity assay

A CCK-8 assay was employed to evaluate the cytotoxicity of PTX against 2 cell lines: the PTX-sensitive SUM159PT and the PTX-resistant SUM159/TXR. Corresponding IC_50_ values were determined as 2.738 μg/mL for SUM159PT cells and 35.61 μg/mL for SUM159/TXR cells (Figure 5a), with a RI of 13.01. By contrast, PSO exhibited comparable IC_50​_ values in SUM159PT and SUM159/TXR cells, which were 63.03 μg/mL and 70.58 μg/mL, respectively (Figure 5b), indicating that SUM159/TXR cells did not develop cross-resistance to PSO.

**FIGURE 5:**
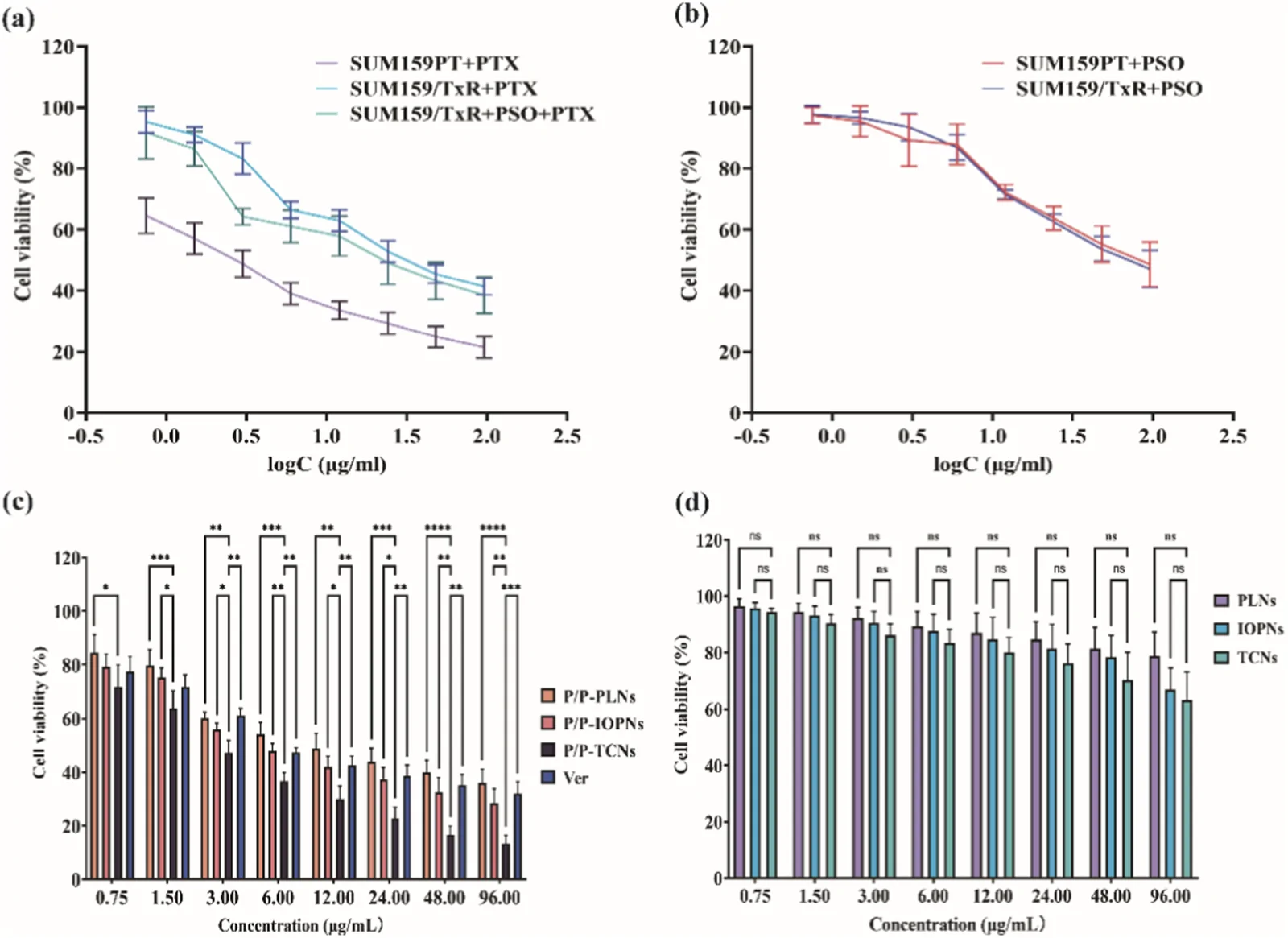
Cytotoxicity. **(a)** Cell viability of SUM159PT cells treated with PTX, SUM159/TXR cells treated with PTX, and SUM159/TXR cells co-treated with PSO and PTX; **(b)** Cell viability of SUM159PT and SUM159/TXR cells treated with PSO alone; **(c)** Cell viability of SUM159/TXR cells following treatment with P/P-PLNs, P/P-IOPNs, P/P-TCNs, and verapamil (Ver) as the positive control; **(d)** Cell viability of SUM159/TXR cells treated with blank carriers PLNs, IOPNs and TCNs. Data are presented as mean ± SD (n = 6). **** P < 0.0001, *** P < 0.001, ** P < 0.01, * P < 0.05 vs. P/P-TCNs (M) group.

To investigate the potential of PSO as a PTX sensitizer in SUM159/TXR cells, a co-treatment regimen of PSO and PTX was implemented. Experimental data indicated that co-administration of the two agents significantly improved the responsiveness of SUM159/TXR cells to PTX, with the PTX IC_50_ decreasing from 35.61 μg/mL to 24.83 μg/mL. This sensitizing effect may be related to PSO’s ability to modulate the tumor microenvironment or reverse the PTX resistance-related pathways (e.g., inhibiting P-glycoprotein-mediated drug efflux), thereby increasing the intracellular accumulation of PTX (Meng et al., 2024).

To further improve the bioavailability of PSO and PTX, reduce their off-target toxicity, and enhance their anti-tumor efficacy in resistant cells, the two drugs were co-encapsulated into P/P-PLNs, P/P-IOPNs and P/P-TCNs, respectively. The IC_50_ values of these three nanoparticles against SUM159/TXR cells were determined to be 14.99 μg/mL, 7.960 μg/mL and 3.050 μg/mL, respectively (Figure 5c). Among the three formulations, P/P-TCNs exhibited the most significant cytotoxicity, which may be attributed to the unique physicochemical properties of TCNs (e.g., high specific surface area, excellent biocompatibility, and efficient intracellular delivery capability) that facilitate drug release and cellular internalization (Eltaib, 2025).

Additionally, the cytotoxic effects of the blank nanoparticle carriers (PLNs, IOPNs and TCNs) were assessed to exclude carrier-related cell damage (Figure 5d). The results showed that the blank carriers had no obvious cytotoxicity against SUM159/TXR cells, indicating that PLNs, IOPNs and TCNs were biocompatible and safe. Collectively, these findings confirmed that P/P-TCNs possessed favorable efficacy and safety for inhibiting the proliferation of SUM159/TXR cells.

### 3.4 *In vitro* cellular uptake

In cultured SUM159/TXR cells, the cellular uptake fluorescence intensity followed the order of C6-TCNs > C6-PLNs > C6-IOPNs > C6 (Figures 6a–c). These results indicated that all three prepared formulations could effectively target tumor cells and enhance the cellular uptake of PSO and PTX. The distinct uptake efficiency among different groups is presumed to be associated with the inherent properties of nanoparticles (Shanahan et al., 2025).

**FIGURE 6:**
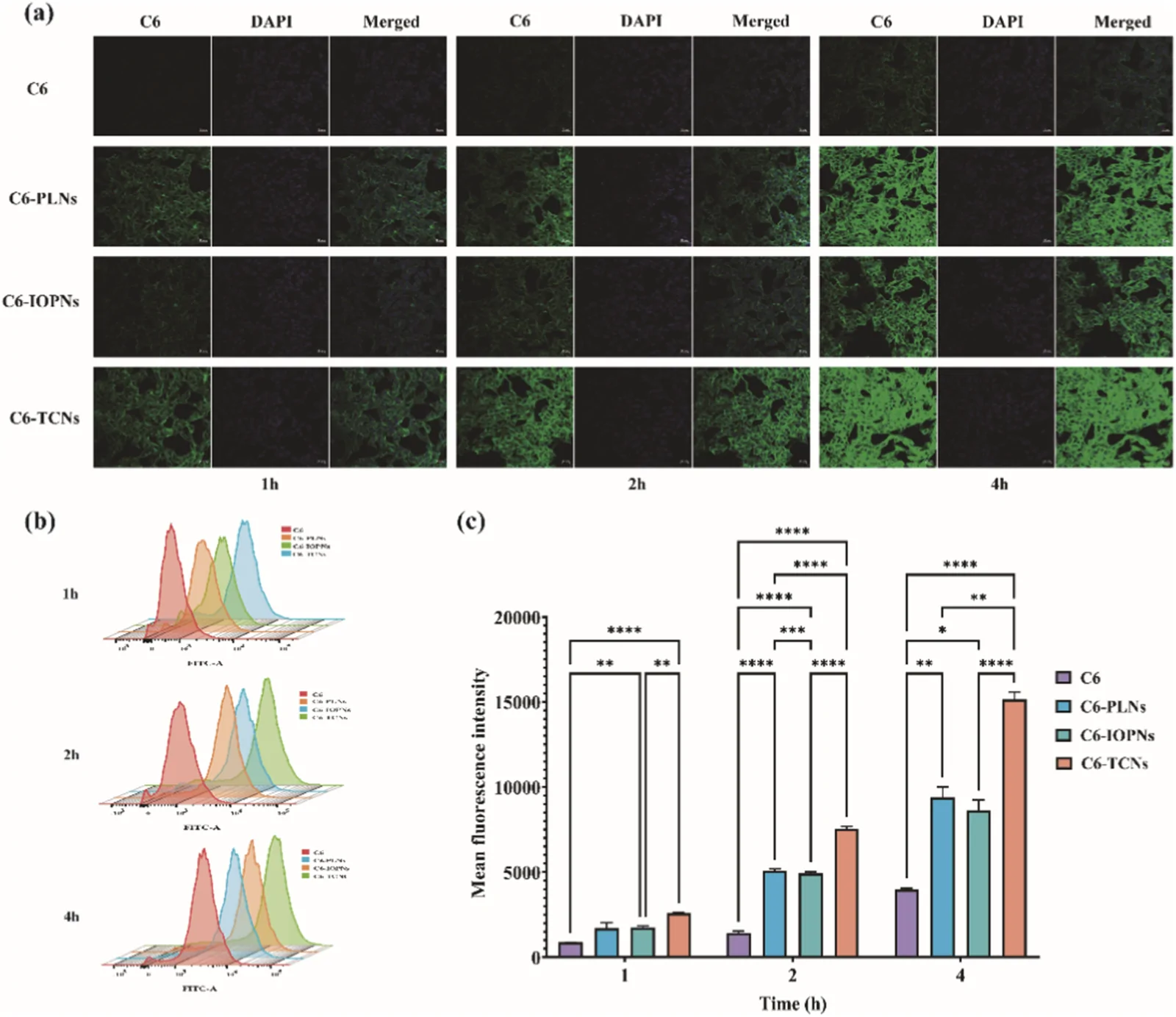
Cellular Uptake Efficiency of Nanoparticles. **(a)** Laser confocal microscopy images of SUM159/TXR cells exposed to free C6, C6-PLNs, C6-IOPNs and C6-TCNs, Scale bar = 20 μm; **(b,c)** Flow cytometry fluorescence histograms of SUM159/TXR cells exposed to free C6, C6-PLNs, C6-IOPNs and C6-TCNs. Data are expressed as mean ± SD (n = 3). **** P < 0.0001, *** P < 0.001, ** P < 0.01, * P < 0.05 vs. C6-TCNs group.

### 3.5 Cell cycle analysis

As shown in Figures 7a–e, the results indicated that PSO exerted a predominant G0/G1 phase arrest effect, which inhibited cellular DNA replication. PTX, a classic tubulin inhibitor, mainly binds to tubulin to suppress microtubule depolymerization, disrupt the structure of the mitotic spindle, and thus block cells from undergoing normal mitosis, ultimately inducing G2/M phase arrest. A large number of scientific studies have verified this mechanism of action of PTX (Sati et al., 2024). The combined treatment of PSO and PTX resulted in a mild concurrent G0/G1 and G2/M phase arrest. PLNs had no impact on the cell cycle progression, while IOPNs and TCNs induced a slight G2/M phase arrest. P/P-PLNs, P/P-IOPNs, P/P-TCNs (L) and P/P-TCNs (M) exhibited comparable G0/G1 phase arrest effects; however, a notable difference was that P/P-TCNs (L) and P/P-TCNs (M) showed a stronger G2/M phase arrest effect. At a high dose, P/P-TCNs exerted a robust G2/M phase arrest effect. Compared with the P/P group, the three nanoformulations not only exerted a significant G0/G1 phase arrest effect but also enhanced the G2/M phase arrest effect of PTX, such improved cell cycle regulation ability further verified the advantages of the constructed nano-delivery systems for anti-tumor combination therapy.

**FIGURE 7:**
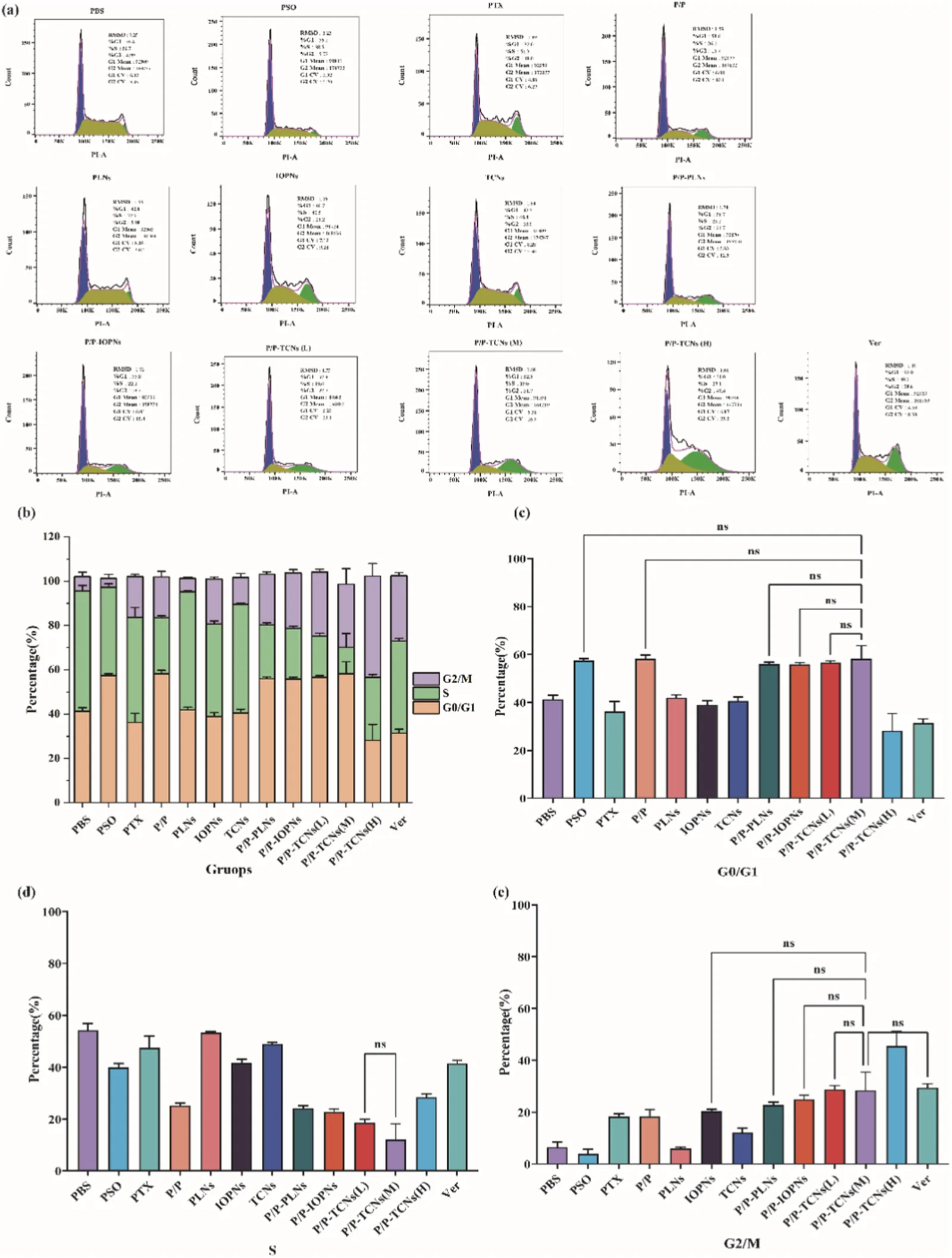
Cell cycle analysis. **(a)** Flow cytometry-based cell cycle profiling; **(b)** Cell cycle phase distribution; **(c)** G0/G1 phase cell proportion; **(d)** S phase cell proportion; **(e)** G2/M phase cell proportion. Data are expressed as mean ± SD (n = 3). ****P < 0.0001, ***P < 0.001, **P < 0.01, *P < 0.05 vs. P/P-TCNs(M) group. In Figure c, d and e, only the comparisons with no significant difference (ns) are labeled; all other comparisons are statistically significant.

The differential cell cycle arrest effects of PSO, PTX and their combined nanoformulations can be elaborated as follows. PSO induces G0/G1 phase arrest by regulating key molecules at the G1/S checkpoint, inhibiting cyclin-CDK complex activity to block Rb phosphorylation and DNA replication-related gene transcription (Zou et al., 2024). PTX mediates G2/M phase arrest via specific binding to β-tubulin, stabilizing microtubules, disrupting mitotic spindle assembly and trapping cells in the G2/M phase (Wang et al., 2023). Their synergistic arrest effect—targeting both G0/G1 and G2/M checkpoints—covers more tumor cells (proliferating and quiescent), reduces tumor cell escape and enhances antitumor efficacy (Zhao et al., 2023). Meanwhile, mild G2/M phase arrest induced by IOPNs and TCNs and high-dose P/P-TCNs show enhanced G2/M arrest compared with P/P-PLNs may be linked to iron-induced ferroptosis accroding to reported literature (Dixon and Olzmann, 2024).

### 3.6 Cell apoptosis assay

As shown in Figures 8a,b, the apoptosis assay revealed that the apoptotic rate exhibited the following order: P/P-TCNs (H) > P/P-TCNs (M) > P/P-TCNs (L) > P/P-IOPNs ≈ Ver > P/P-PLNs > P/P > PTX > PSO. Medium and high doses of P/P-TCNs induced significantly higher apoptosis than the free P/P combination (p < 0.05), and this effect exhibited a clear dose-dependent manner. Notably, nanoformulations tended to induce more early apoptosis. The proportion of late apoptotic cells was comparable between most nano groups and the P/P group. The P/P-TCN system exerted superior pro-apoptotic activity. The pro-apoptotic effect of P/P-TCNs was positively correlated with its working concentration. Combined with the apoptosis distribution results, we found that P/P-TCNs and free PTX differed in their apoptotic phenotypes. Combined with the superior cellular uptake of P/P-TCNs observed above, increased intracellular drug content is likely one factor contributing to its enhanced apoptotic effect. Relevant previous studies have discussed the relationships among drug concentration, apoptosis regulation and tumor drug resistance (Bender et al., 2025; Teng et al., 2026).

**FIGURE 8:**
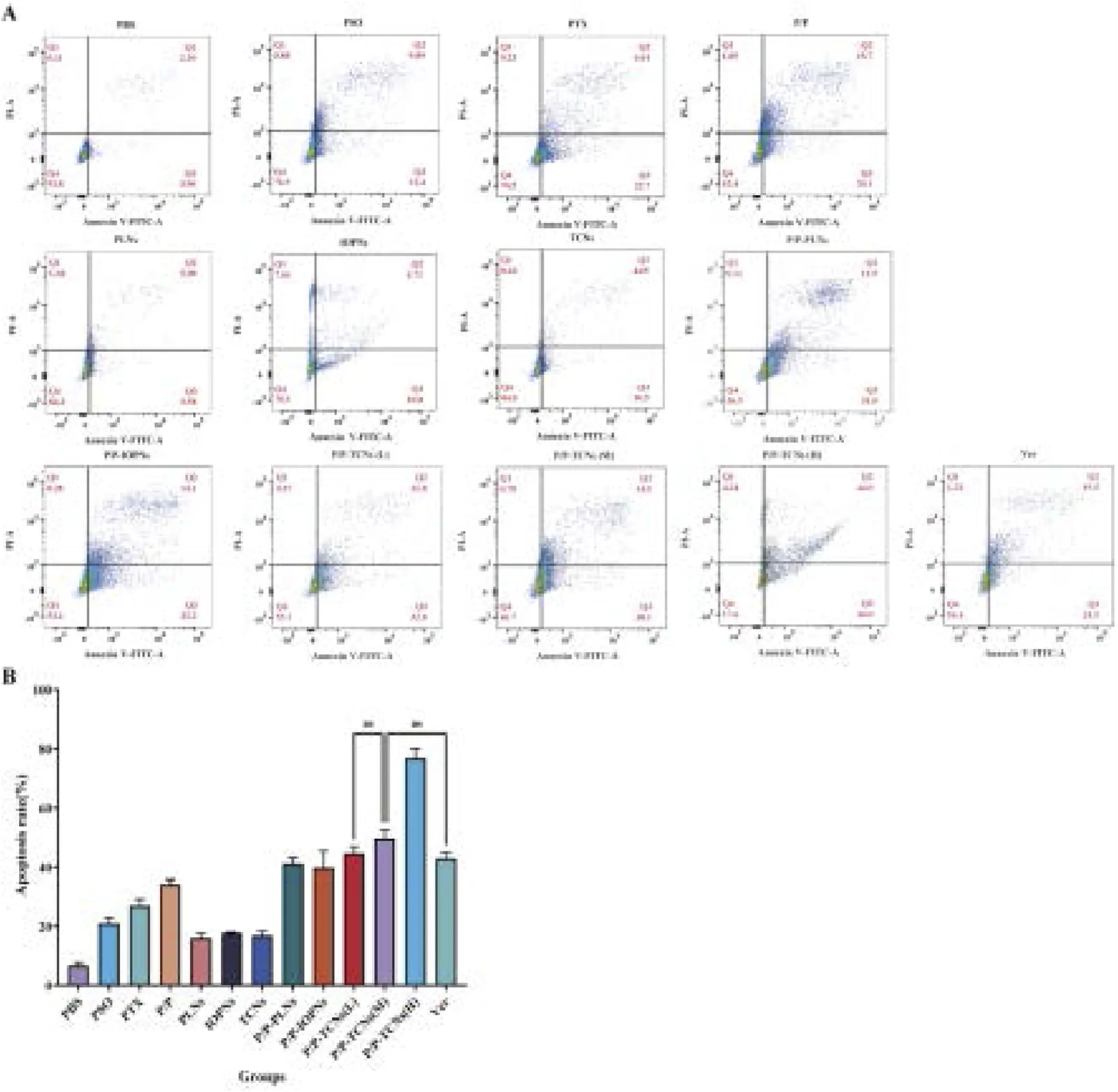
Cell Apoptosis. **(A)** Apoptosis analysis by flow cytometry; **(B)** Apoptotic rate. Data are expressed as mean ± SD (n = 3). ****P < 0.0001, ***P < 0.001, **P < 0.01, *P < 0.05 vs. P/P-TCNs (M) group. In Figure b, only the comparisons with no significant differences are labeled, all other comparisons are statistically significant.

Inducing tumor cell apoptosis is a core strategy for antitumor therapy. Our results demonstrated that the constructed nano-delivery systems, especially P/P-TCNs, could effectively enhance the pro-apoptotic ability of PSO and PTX. The dose-dependent effect enables flexible regulation of therapeutic efficacy. Combined with higher cellular uptake, these nanoformulations show great potential for improving the antitumor effect of combined medication.

### 3.7 qPCR detection of ABCB1 mRNA levels

As shown in Figure 9, PBS-treated paclitaxel-resistant SUM159/TXR triple-negative breast cancer cells displayed high basal ABCB1 mRNA expression. Free PTX treatment further upregulated ABCB1 transcription, consistent with the known ability of paclitaxel to induce P-gp expression and reinforce drug resistance in resistant tumor cells. Free PSO alone slightly decreased ABCB1 mRNA, whereas the free combination of PSO and PTX only led to a mild reduction. Groups treated with blank carriers (PLNs, IOPNs and TCNs) showed minimal inhibition of ABCB1 mRNA expression, verifying that the carrier materials barely affect ABCB1 transcription. In comparison, PSO/PTX co-loaded TCNs nanoparticles markedly downregulated ABCB1 mRNA expression in a dose-dependent fashion. Collectively, these findings suggest that P/P-TCNs nanoparticles suppress ABCB1 transcription, thereby reversing PTX-associated multidrug resistance.

**FIGURE 9:**
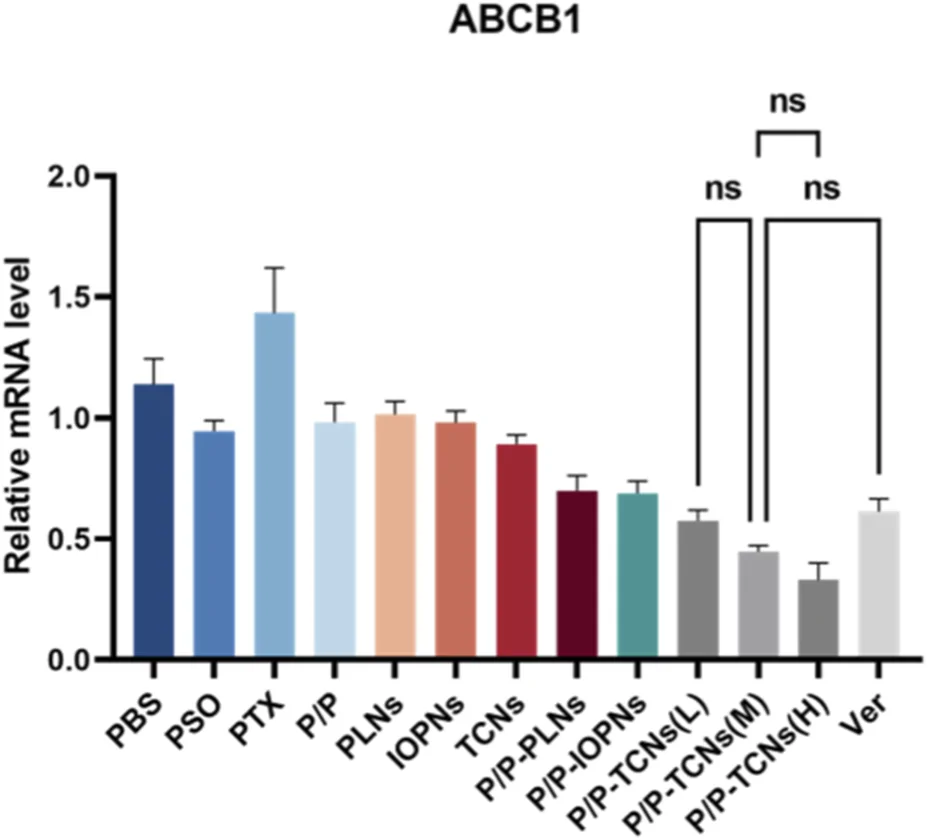
ABCB1 mRNA levels. Data are expressed as mean ± SD (n = 3). ****P < 0.0001, ***P < 0.001, **P < 0.01, *P < 0.05 vs. P/P-TCNs (M) group. Only the comparisons with no significant differences are labeled, all other comparisons are statistically significant.

### 3.8 *In vivo* biodistribution

*In vivo* fluorescence imaging in Figure 10 showed distinct biodistribution differences among various formulations in paclitaxel-resistant TNBC tumor-bearing mice. Compared with free ICG, all nanoparticle groups exhibited prolonged *in vivo* circulation and slower metabolic clearance, retaining longer-term fluorescent signals in tumors. For magnetic ICG-IOPNs and ICG-TCNs formulations, the groups treated with an external magnetic field displayed earlier tumor accumulation and significantly higher intratumoral fluorescence intensity than the non-magnetic groups. These results confirmed that the prepared magnetic nanoparticles possessed magnetic-targeting accumulation ability under external magnetic guidance.

**FIGURE 10:**
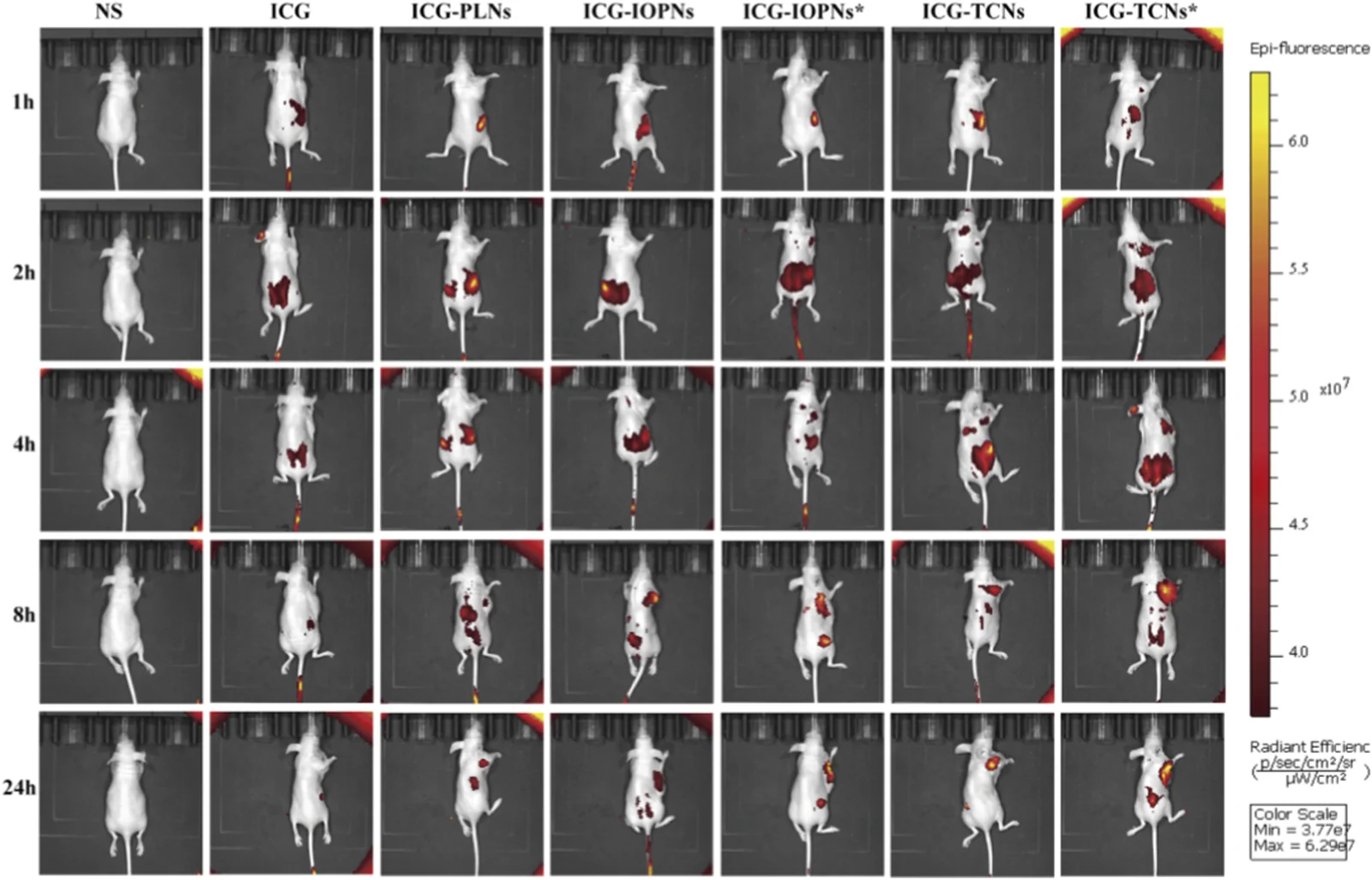
Boidistribution of ICG-labeled formulations in the tissues of BALB/c nude mice at 1, 2, 4, 8, 24 h post-injection. Asterisks (*) in the figure denote groups with external magnetic field treatment.

### 3.9 *In vivo* anti-tumor efficacy

Finally, the *in vivo* antitumor effects of P/P-PLNs, P/P-IOPNs, P/P-TCNs and Ver were tested in xenograft tumor-bearing nude mice. As shown in Figures 11a–d, the tumor inhibition potency followed the sequence: P/P-TCNs > P/P-PLNs ≈ P/P-IOPNs > Ver. Specifically, the medium-dose group of P/P-TCNs achieved a tumor inhibition rate of 80.6% (P < 0.05, compared with the other two nanoparticle formulations). When contrasted with both the PTX monotherapy cohort (tumor inhibition rate of 42.0%) and the PSO-PTX combined treatment cohort (tumor inhibition rate of 48.0%), the medium-dose P/P-TCNs formulation exhibited a marked enhancement in tumor suppressive efficacy. These findings confirmed the effectiveness of P/P-TCNs in reversing tumor multidrug resistance. As evidenced by the histopathological analysis results presented in Figure 11e, no notable pathological changes were observed in any of the treatment cohorts, indicating that the fabricated nanoparticles possessed good biocompatibility and safety characteristics.

**FIGURE 11:**
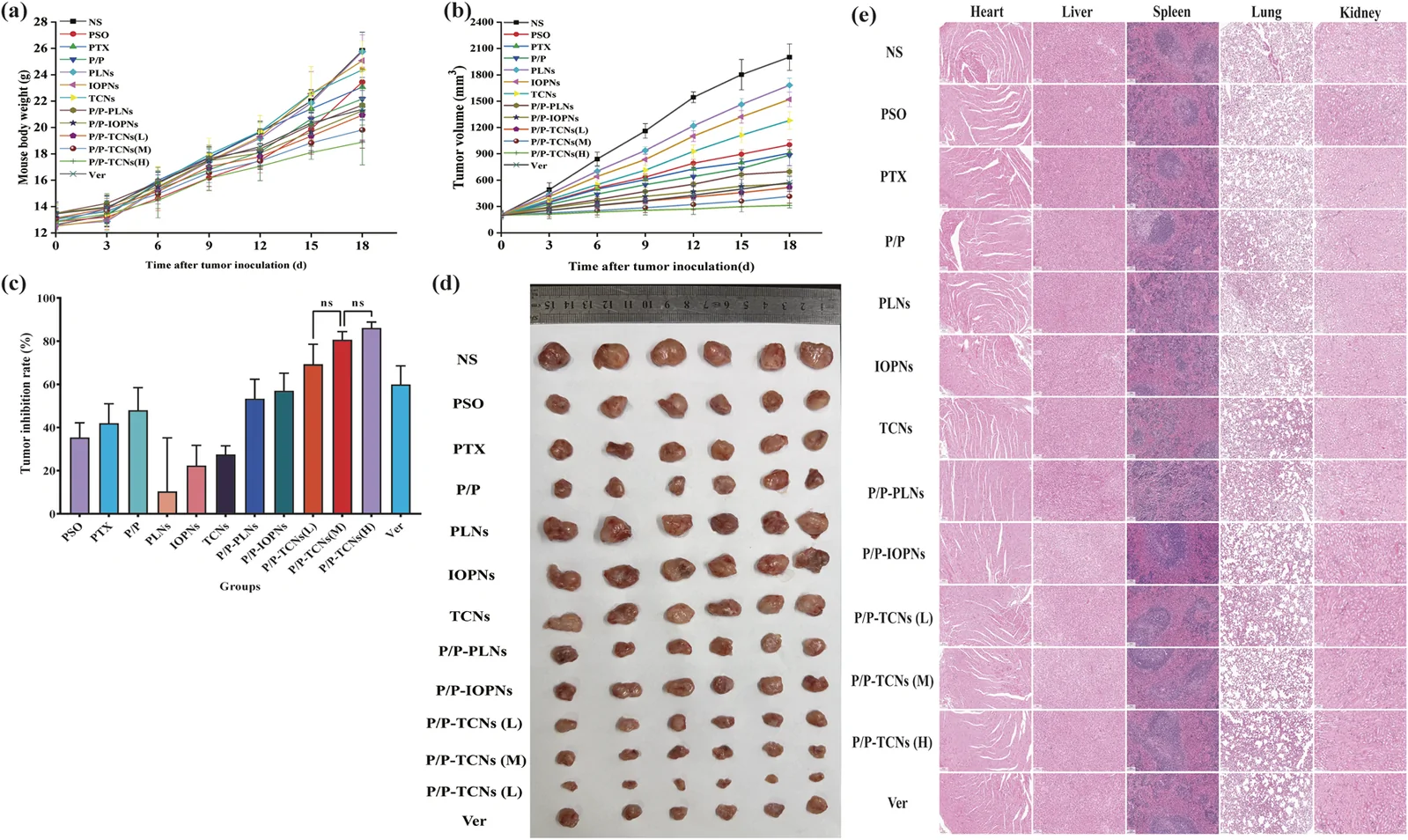
*In Vivo* Anti-Tumor Efficacy and Histological Sections of Major Organs. **(a)** Tumor volume changes in nude mice during the experimental period; **(b)** Tumor weight of nude mice in each group; **(c)** Tumor inhibition rate of each treatment group; **(d)** Representative images of tumor tissues from nude mice after euthanasia; **(e)** Histological Sections of Major Organs. Sequential order (left to right): heart, liver, spleen, lung, kidney. *Data are presented as mean ± SD (n = 6). ****P < 0.0001, ***P < 0.001, **P < 0.05, *P < 0.05 vs. P/P-TCNs(M) group*.* In Figure c, only the comparisons with no significant difference (ns) are labeled; all other comparisons are statistically significant.

The *in vivo* results further validated the application potential of the prepared nanoformulations. Among all groups, P/P-TCNs achieved the strongest antitumor efficacy against drug-resistant tumors, meanwhile maintaining satisfactory biosafety. Combined with *in vitro* findings, this nano delivery system shows great prospect for the treatment of TNBC.

## 4 Conclusion and discussion

In this study, oleic SPIONs@OA were synthesized and co-encapsulated with PSO and PTX into PLNs to prepare P/P-TCNs, a novel nanocomplex designed to reverse PTX resistance in TNBC. Compared with P/P-PLNs, P/P-IOPNs and positive control Ver, P/P-TCNs showed superior biological performance. *In vitro* assays revealed that P/P-TCNs significantly enhanced PTX cytotoxicity against resistant SUM159/TXR cells, with an IC_50_ of 3.050 μg/mL—sharply lower than free PTX’s 35.61 μg/mL—while the carrier exhibited good biocompatibility. Cellular uptake experiments confirmed efficient internalization of P/P-TCNs by tumor cells. Cell cycle analysis demonstrated concentration-dependent arrest: low/medium doses induced dual G0/G1 and G2/M phase arrest, whereas high doses exerted robust G2/M arrest. Apoptosis assays indicated dose-dependent pro-apoptotic effects, with late apoptosis dominant at high doses. *In vivo* xenograft experiments showed that medium-dose P/P-TCNs achieved a tumor inhibition rate of 80.6%.

Fe_3_O_4_-based magnetic nanoparticles have been extensively applied in clinical settings, with substantial clinical evidence supporting their safety and translational value. Specifically, Feraheme® (ferumoxytol), a polymer-functionalized Fe_3_O_4_ nanoparticle approved by the U.S. FDA in 2009, has accumulated abundant clinical data confirming its favorable long-term biosafety and metabolic stability in human subjects. Other clinically available magnetic nanoparticle formulations, such as Resovist® and NanoTherm®, further validate that hydrophilic polymer-modified Fe_3_O_4_ nanoparticles possess mature clinical translation prospects for cancer therapeutic applications. Consistent with these findings, previous studies have also demonstrated the good biodegradability and low immunogenicity of such magnetic nanoparticles, which collectively supports the long-term safety profile of our hybrid nanoparticle system (Tran et al., 2022; Qiao et al., 2023; Yang et al., 2023). Notably, all excipients employed in our formulation, including phospholipids, PLGA and DSPE-PEG2000, and Pluronic F-127, are recognized as biocompatible materials by the FDA and are widely used in clinical practice. In terms of manufacturing, the nanoparticles were fabricated via the emulsion-solvent evaporation technique, a scalable formulation strategy that is analogous to those adopted for two clinically approved nanotherapeutics, Abraxane (FDA-approved in 2005) and Fyarro (FDA-approved in 2021). This similarity in manufacturing processes implies good feasibility for industrial scale-up and subsequent clinical translation. Furthermore, paclitaxel, the chemotherapeutic agent loaded in our nanoplatform, is a well-established standard first-line regimen for triple-negative breast cancer, which further reinforces the clinical relevance and translational potential of the present delivery system.

Overall, this integratedas a promising nanodelivery system, P/P-TCNs delivery system achieves synergistic effects of magnetic targeting, chemotherapy and drug-resistance reversal, providing a novel strategy for precise therapy of in TNBC.

Despite the promising antitumor performance of the constructed functionalized magnetic ternary hybrid nanoparticles, the present study still possesses several inevitable limitations that need to be addressed in further research. First and the most importment, the current mechanism exploration stays at phenotypic observation level. The conclusions concerning multidrug resistance reversal lack direct molecular evidence such as protein quantification and pathway inhibition assays, and relevant mechanistic interpretation remains speculative. Second, the *in vivo* study is incomplete. Pharmacokinetic profile, and tissue distribution have not been performed. The biosafety evaluation merely relies on histopathological observation, without supporting hematological and biochemical detection data. On the basis of the above deficiencies, our follow-up work will focus on clarifying molecular-level mechanism verification, and *in vivo* pharmacology and targeting tests, so as to steadily promote the clinical application potential of this dual-drug co-delivery magnetic nanoplatform.

## Data Availability

The original contributions presented in the study are included in the article/supplementary material, further inquiries can be directed to the corresponding authors.
